# Overview and Critical Appraisal of Arterial Spin Labelling Technique in Brain Perfusion Imaging

**DOI:** 10.1155/2018/5360375

**Published:** 2018-05-08

**Authors:** Amirah Alsaedi, David Thomas, Sotirios Bisdas, Xavier Golay

**Affiliations:** ^1^Department of Radiology Technology, Taibah University, Medina, Saudi Arabia; ^2^Department of Brain Repair & Rehabilitation, UCL Institute of Neurology, London, UK; ^3^National Hospital for Neurology and Neurosurgery, London, UK

## Abstract

Arterial spin labelling (ASL) allows absolute quantification of CBF via a diffusible intrinsic tracer (magnetically labelled blood water) that disperses from the vascular system into neighbouring tissue. Thus, it can provide absolute CBF quantification, which eliminates the need for the contrast agent, and can be performed repeatedly. This review will focus on the common ASL acquisition techniques (continuous, pulsed, and pseudocontinuous ASL) and how ASL image quality might be affected by intrinsic factors that may bias the CBF measurements. We also provide suggestions to mitigate these risks, model appropriately the acquired signal, increase the image quality, and hence estimate the reliability of the CBF, which consists an important noninvasive biomarker. Emerging methodologies for extraction of new ASL-based biomarkers, such as arterial arrival time (AAT) and arterial blood volume (aBV), will be also briefly discussed.

## 1. Introduction

Histopathologic assessment is considered to be the gold standard for brain tumour diagnosis. However, it suffers from several limitations including sampling errors and its dependence on invasive biopsies or surgical removal of a tumour [1]. Imaging modalities in clinical settings can therefore make a great contribution towards determining the most appropriate treatment. In particular, MRI plays a leading role in all phases of tumour management, including diagnosis, therapy, and follow-up due to the noninvasive nature and sensitivity to soft tissue changes. Conventional MRI images help us to determine tumour morphology and the involvement location among brain tissue of normal appearance by using imaging contrasts such as *T*1 and *T*2 weighted images (*T*1-W and *T*2-W, resp.), while fluid-attenuated inversion recovery (FLAIR) provides information regarding oedema. Additionally *T*1-W postcontrast allows the clinician to identify any regions of *blood brain barrier* (BBB) disruption [2, 3]. However, some low-grade gliomas demonstrate contrast uptake, while a lack of enhancement may be found in some high-grade gliomas [4].

Tumour growth beyond 1 to 2 cm in diameter depends on vessel formation (angiogenesis) to supply blood and nutrition to the tumour tissue. This angiogenesis plays a critical role in tumour survival, development, and malignant transformation [5, 6]. Additionally, angiogenesis reflects the tumour type [7] and they are a key element of tumour pathophysiology [8]. Cerebral perfusion at the capillary level yields accurate information about tumour neoangiogenesis [9–11]. This, known as *cerebral blood flow* (CBF), refers to the delivery of blood volume to an element of tissue at the capillary level in a given period of time (ml/100 gm/min).

Absolute quantification of CBF requires a diffusible tracer that disperses from the vascular system into neighbouring tissue. However, an intravascular contrast injection (nondiffusible), while unable to absolutely quantify CBF, can infer it by using the central volume principle [12, 13] (cerebral blood flow calculated as a ratio between blood volume in a given volume of tissue and the average time for the tracer to pass through this volume). Both diffusible and intravascular tracers reveal haemodynamic measurements known collectively as perfusion imaging. Perfusion MRI is a functional imaging method that produces advanced image contrast, and such images provide quantitative measurements that reflect relevant functional characteristics such as CBF and *cerebral blood volume* (CBV). These measurements mirror the regional hemodynamic of the microvasculatures, which in turn represents the cerebral metabolic demand [14] such as tumour pathophysiology [8]. For example, glioma progression has been indicated to be strongly reliant on such angiogenesis [7], so high perfusion rates reflect tumour malignancy [7]. So, perfusion MRI methods can provide information regarding angiogenesis.

The three most common perfusion MRI methods are *dynamic susceptibility contrast* (DSC), which exploits the magnetic field-susceptibility caused by the first pass of a high concentration of an extrinsic contrast agent using dynamic T2, *dynamic contrast enhancement* (DCE), which also uses an extrinsic contrast agent followed by dynamic *T*1-W, and *arterial spin labelling* (ASL), which uses magnetically labelled blood water as an inherently diffusible tracer. ASL takes advantage of using the arterial blood water as an inherent diffusible tracer, subsequently, offering a noninvasive imaging method that generates absolute cerebral blood flow quantification. ASL, as suggested by previous studies, could be a reliable alternative to other invasive perfusion techniques such as DSC [15, 16] and *positron emission tomography* (PET) [17, 18]. This review therefore focuses on the methodological background of ASL and its application in brain tumours.

## 2. ASL Main Principle

**Figure 1:**
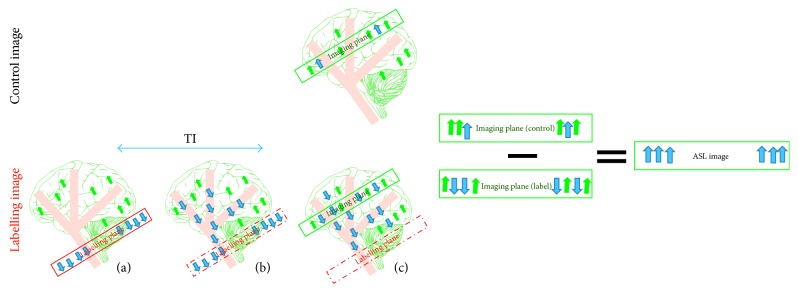
The drawing shows the main mechanisms of arterial spin labelling (ASL). The labelling image is acquired using blood water as a diffusible tracer. (a) The proximal labelling plane to the imaging target point where the flowing protons are magnetically labelled via inversion (blue arrows); (b) during a delay time, TI, the tagged blood water leaves the label plane and starts to disperse into tissue at the image plane; (c) the labelled image is obtained. The control image is acquired without a labelling pulse in order to extract the tagged blood water from the static tissue (green arrows). Subtracting the two images (control−labelled) leaves the tagged protons, which are directly proportional to the tissue perfusion.

The main principle of the ASL technique is shown in Figure 1, and its goal is to quantify CBF or the blood delivery rate to a regional voxel of brain tissue in the target image slice. Thus, a 180 RF pulse is applied at the labelling plane, which is upstream of the target image slice, to invert the blood water magnetisation. The tagged blood water flowing into the brain tissue is proportional to the CBF. After an appropriate delay time, also known as inversion time or postlabelling delay time (TI/PLD), the labelled magnetisation reaches the slice of interest in a period known as arterial arrival time or transit time (AAT), and the image slice is acquired (raw data). In order to extract the perfusion information, the same experiment is then repeated without inversion of the blood water magnetisation. After the same delay time, TI, the control image is obtained. By subtracting the static tissue through the difference between the tagged image and the control image, the ASL difference image is created. The difference signals reflect the tagged blood water magnetisation that arrived to the image slice during TI, and hence these signals are directly proportional to the CBF [19].

In an ideal ASL experiment, within the control image, the blood water magnetisation is fully relaxed (*M*_0_*a*). However, following the application of the inversion pulse, the magnetisation (−*M*_0_*a*) brought to the labelled image by the local blood flow (*f*) during time delay (*TI*) is

(1) $$\begin{array}{c} -M_{0}a*f*TI. \end{array}$$

The difference signal between the longitudinal magnetisation of the control and label images (Δ*M*) is

(2) $$\begin{array}{c} \Delta M=2M_{0}a*f*TI. \end{array}$$

In the case of imaged tissue where *TI* ≈ 1 sec, and 100 ml of tagged blood volume is delivered in a normal flow rate of 60 ml/min, the magnetisation variation will be around 1%. This example reflects the main drawback of ASL images, which is their weak signal-to-noise ratio (SNR). This problem will be discussed latter. Moreover, since the signal difference is around 1%, it is important that this is connected to the perfusion signals rather than any parasitic signals. Both tagged and control images should therefore have identical static tissue signals for successful measurement.

## 3. CBF Quantification Models

ASL images are usually provided as raw data (source images). These images must undergo computation processes to provide a CBF map. This map then subsequently undergoes qualitative and/or quantitative evaluation. It is important to utilise a detailed model that combines the process of kinetics and relaxation, in order to extract a reliable quantitative measurement from the ASL data.

### 3.1. *T*1 Model

A modification of the Bloch equation for longitudinal magnetisation to include the flow effect according to Fick's principle (difference between delivery and clearance of substance in blood is proportional to the local flow) has been introduced by Detre and Williams [19]:

(3) $$\begin{array}{c} \frac{dM\left(t\right)}{dt}=\frac{M_{0}-M\left(t\right)}{T1}+fMa\left(t\right)-fMv\left(t\right), \end{array}$$

where *M*_0_ is the longitudinal magnetisation of the tissue at the equilibrium and *M*, *M*a, and *Mv* are the time-dependent longitudinal magnetizations of the tissue, arterial, and venous blood, respectively. This is known as the *T*1-quantification model [20]. As previously mentioned, the ASL difference image reflects the concentration of the delivered magnetisation. This difference from the equilibrium will help us to see the impact of flow on the apparent relaxation time *T*1  app. Thus, it can be assumed that the flow delivers magnetisation:

(4) $$\begin{array}{c} \left(fM_{0}adt\right), \end{array}$$

as well as clearing it

(5) $$\begin{array}{c} \left(fMvdt\right), \end{array}$$

and that these actions occur concurrently. Furthermore, by considering two assumptions, the relationship between the magnetisation of blood and tissue can be clarified. First, brain tissue can be considered as a well-mixed compartment, given by

(6) $$\begin{array}{c} Mv\left(t\right)=M\left(t\right)/\lambda , \end{array}$$

where *λ* is the volume distribution between tissue and blood, which describes an equilibrium distribution of tracer between the tissue and blood. The second assumption, fully relaxed blood magnetisation *M*_0_*a* during equilibrium equals to *M*_0_/*λ*; thus, the magnetisation change because of flow from (4–6) is given by

(7) $$\begin{array}{c} dM\left(\text{flow}\right)=fM_{0}a\left(t\right)-fMv\left(t\right)=\left(M_{0}-M\left(t\right)\right)\frac{fdt}{\lambda }. \end{array}$$

Applying (7) to (3) gives

(8) $$\begin{array}{c} \frac{dM\left(t\right)}{dt}=\frac{M_{0}-M\left(t\right)}{T1}+\frac{f}{\lambda }\left(M_{0}-M\left(t\right)\right). \end{array}$$

Additionally, labelled blood flow leads to a time-dependent decrease in the longitudinal magnetisation *M*_0_ with an apparent time constant *T*1  app; its magnitude depends on the *T*1 relaxation time of tissue and blood flow, given by

(9) $$\begin{array}{c} \frac{1}{T1\text{ app}}=\frac{1}{T1}+\frac{f}{\lambda }, \end{array}$$

and in the case of a steady state, *M*_0_ decrease to longitudinal magnetisation in steady state *M*_ss_, and the solution is given by

(10) $$\begin{array}{c} f=\frac{\lambda }{T1\text{ app}}\left(\frac{M\text{control}-M\text{label}}{2M\text{control}}\right). \end{array}$$

### 3.2. General Kinetic Model

The previous model did not include any potential systematic error in ASL. These errors can arise for several reasons, including variable transit time, exchanges of tagged water between the capillaries and tissue and its impact on the *T*1 relaxation time, and incomplete tagged water extraction from the capillaries. A more general model that is flexible enough to include the potential systematic error in ASL can be created based on the general kinetic model [21]. This model considers the difference signal (Δ*M*) as a concentration of a tracer (magnetisation) that is delivered by flow and remains in the tissue at time *t*. The amount of remaining magnetisation at time *t* will depend on the delivery of the magnetisation by the arterial flow and clearance via venous flow and longitudinal magnetisation. To describe the process of delivery and clearance, three functions time have to be defined:

- The delivery function, *C*(*t*), is the amount of magnetisation arriving the voxel at time *t*.
- The residue function, *R*(*t*), is the fraction of the remaining tagged water at time *t* after arrival in the voxel.
- And the magnetisation relaxation function, *M*(*t*), is the fraction of the remaining longitudinal magnetisation that is carried by the tagged water at time *t* after arrival.

**Figure 2:**
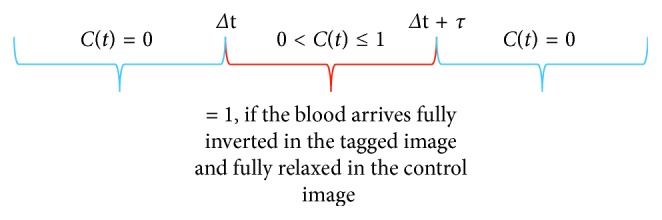
A drawing showing the delivery function *C*(*t*), which is not zero at Δ*t*+*τ*Δ*t* < *t* < Δ*t*+*τ*.

Here, the potential systematic error in ASL can be taken into account by using the appropriate form of these time functions. In order to define the appropriate form of the functions, three key assumptions are required for the standard model. The first assumption, as displayed in Figure 2, is that before the initial transit time (Δ*t*), no labelled blood arrives. In contrast, after Δ*t* and as the tagged blood continues to arrive as long as the duration of the arterial bolus (Δ*t*+*τ*), the delivery function is given by

(11) $$\begin{array}{c} \begin{array}{l} C\left(t\right)=e^{-\left(t/T1b\right)},\text{ PASL and one single pulse,}\\ C\left(t\right)=e^{-\left(\Delta t/T1b\right)},\text{ CASL and long pulse}. \end{array} \end{array}$$

The second assumption is that the kinetics of the water exchange between the tissue and blood are assumed to follow the rules of “single-compartment kinetics”. In other words, there is a rapid exchange between any subcompartments that exist among these tissues, leading to a constant tissue concentration as a time function. In particular, by assuming a constant concentration ratio between tissue and venous areas which is equal to the tissue/blood water partition coefficient, the residue function is given by

(12) $$\begin{array}{c} r\left(t\right)=e^{-ft/\lambda }. \end{array}$$

The third assumption is that, immediately after the tagged water molecules arriving the voxel, they are totally extracted from the vascular space so that the tagging will decay with the relaxation time *T*1 of the tissue, given by

(13) $$\begin{array}{c} m\left(t\right)=e^{-t/T1}. \end{array}$$

Thus, the standard general kinetic model from (11–13) is

(14) $$\begin{array}{c} \begin{array}{l} C\left(t\right)=\left\{\begin{array}{ll} 0 & 0<t<\Delta t\\ e^{-t/T1b}\text{PASL} & \\ e^{-\Delta t/T1b}\text{CASL} & \Delta t<t<\Delta t+\tau \\ 0, & \end{array}\right.\\ r\left(t\right)=e^{-ft/\lambda },\;\Delta t+\tau <t,\\ m\left(t\right)=e^{-t/T1}, \end{array} \end{array}$$

where Δ*t* is the tagged blood initial arrival time, *τ* is the duration of the arterial bolus (bolus width), and Δ*t*+*τ* expresses the continuous delivery of the labelled blood to an imaging slice as long as the bolus width.

Using a modified equation based on indicator-dilution theory [13], for any agent that is not metabolized, the tissue concentration curve *Ct*(*t*) is the convolution (∗) of the arterial concentration curve *C*(*t*) with the local impulse function *R*(*t*):

(15) $$\begin{array}{c} Ct\left(t\right)=fC\left(t\right)*R\left(t\right), \end{array}$$

where the impulse function in ASL includes *r*(*t*) and *m*(*t*). Following the inversion pulse, the arterial magnetisation difference is

(16) $$\begin{array}{c} \Delta M=2M_{0}a. \end{array}$$

which is delivered by flow to the voxel as

(17) $$\begin{array}{c} 2M_{0}afC\left(t\right). \end{array}$$

Thus, a measure of cerebral perfusion using the freely diffusible indicator-dilution theory [21] and applying (17) to (15) is given by

(18) $$\begin{array}{c} \Delta M\left(t\right)=2M_{0}afC\left(t\right)*\left[r\left(t\right)m\left(t\right)\right], \end{array}$$

where ∗ refers to convolution and the brackets denote the integral of the time dimension (duration of the arterial bolus).

**Figure 3:**
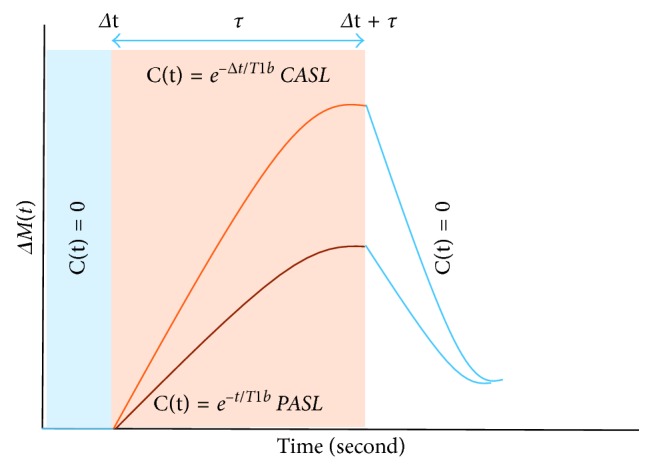
General kinetic model curve illustrating the difference in the delivery bolus *C*(*t*) between CASL and PASL. Where the uppermost curve represents the CASL, and the lower curve represents the PASL. Respectively, the blue, red, and light blue boxes represent the different part of the general kinetic model.

Applying the assumptions from (14) to (18) gives the following solution for PASL:

(19) $$\begin{array}{c} \Delta M\left(t\right)=\left\{\begin{array}{ll} 0, & 0<t<\Delta t\\ \frac{-2M_{0}a\cdot \alpha \cdot f}{\delta R}e^{-R1a\cdot t}\left(1-e^{-\delta R\left(t-\Delta t\right)}\right), & \Delta t<t<\Delta t+\tau \\ \frac{-2M_{0}a\cdot \alpha \cdot f}{\delta R}e^{-R1a\cdot \Delta t}\left(1-e^{\delta R\left(t-\Delta t\right)}\right)\cdot e^{-R1\text{app}\left(t-\left(\Delta t+\tau \right)\right)}, & \Delta t+\tau <t, \end{array}\right. \end{array}$$

where *α* is the inversion efficiency, *R*1*a* is the blood relaxation rate, and *R*1app is the apparent tissue relaxation rate where *δR*=*R*1*a* − *R*1app. Each part of the solution of the general kinetic model takes place over a specific duration as displayed on the curve in Figure 3.

Quantitative perfusion estimates based on ASL can be derived from several models. For simplicity, the kinetic model that is shown before has been proposed in the white paper [22] with certain specific assumptions that quantify the CBF from the ASL raw data at a single delay time. Those three main assumptions are as follows. First, the whole magnetisation bolus (tagged blood) received by the tissue of interest as long as the delay time is longer than the arterial arrival time (AAT). Second, there is retention of the bolus within the tissue (no outflow condition) due to the large water pool. Third, the magnetisation of the labelled blood water decreases due to *T*1*a*. Although the last assumption is not strictly true, the related error is typically relatively small because of the small difference between the *T*1 relaxation of the tissue and blood. Subsequently, the CBF can be quantified in each voxel for PCASL using

(20) $$\begin{array}{c} \text{CBF}=\frac{6000\cdot \lambda \cdot \left(\text{SI control}-\text{SI label}\right)\cdot e^{\text{PLD}/T1a}}{2\cdot \alpha \cdot T1a\cdot \text{SIPD}\cdot \left(1-e^{-\tau /T1a}\right)}\left[\text{ml/}100\text{ g/min}\right]. \end{array}$$

The equation for QUIPSS II PASL is

(21) $$\begin{array}{c} \text{CBF}=\frac{6000\cdot \lambda \cdot \left(\text{SI control}-\text{SI label}\right)\cdot e^{TI/T1a}}{2\cdot \alpha \cdot TI1\cdot \text{SIPD}}\left[\text{ml/}100\text{ g/min}\right], \end{array}$$

where this calculation contains constants values, measured values for each subject, and fixed durations for each acquisition. The constant values are a factor of 6000, used to convert the CBF unit of ml/g/s to ml/100 g/min; *λ* is the water blood/water partition coefficient, determined as 0.9 ml/g; *α*, which is the labelling efficiency and depends on the labelling approach used (for PCASL, *α* = 0.85, and for PASL, *α* = 0.98) [22]; and *T*1*a* is the blood relaxation time, which is approximately equal to 1650 ms at 3 tesla [23]. The measured values, SI  control and SI  label, are the average signal intensity of the control and label images, respectively. Additionally, a measured value of proton density signal intensity, SIPD is used as a scaling image for the ASL difference image to allow an absolute CBF quantification because it provides fully relaxed signal intensity of blood. The fixed duration values are the labelling duration *τ* which for the PCASL recommended to be 1800 ms, while for the PASL is recommended to be 15 to 20 cm tagging slab thickness. The delay time for PCASL is therefore PLD, and for the QUIPSS II PASL, it is *TI*.

## 4. Arterial Spin Labelling Approaches

ASL uses adiabatic pulse instead of standard RF pulses. Standard RF pulse is applied orthogonally to the main magnetic field (static field (B0)) and uses fixed frequency opposed to adiabatic pulse which is a class of RF pulse that is frequency and amplitude modulated as a function of time. In brief as the spins under the static field (B0) precess at their resonance frequency (*f*0), an RF field (B1) is applied in a gradual manner until frequency and amplitude change as a function of time (from below-resonance to resonance and beyond-resonance). In this way, the resulting magnetisation vector is inverted. This pulse type has advantages in terms of tolerance B0 inhomogeneity, and it is less sensitive to B1 miscalibration. In addition, adiabatic inversion helps us to create a sharp slice profile and hence allows better determination and a clear arterial bolus width [24].

**Figure 4:**
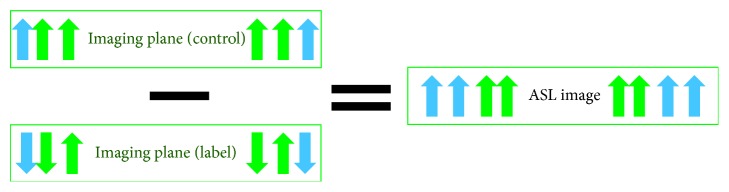
Impact of the MT effect on perfusion evaluation. This effect causes a reduction in the static tissue in the labelling image leading to a subsequent overestimating of perfusion quantification. Static tissue is represented by green arrows and tagged blood water by blue arrows.

As mentioned above the static spins have to be the same among both labelled and control images in order to extract the CBF precisely. The longitudinal magnetisation of the static spins in the label image is, however, affected by the off-resonance pulse through the magnetisation transfer (MT) effects (Figure 4). Subsequently, the labelled image will have less static spins than the control image. Accordingly, ASL difference images contain biased signals from the static tissue, which results in CBF overestimation. To overcome this, the control images must produce the same MT effects without retagging the arterial blood water. This is achieved by applying the same inversion pulse distally to the slice of interest. In this case, the MT effects in the control image cancel out the MT effects that are induced in the labelling image by the labelling pulse [25–27].

The ASL labelling approach can be categorized into two main types: continuous arterial spin labelling (CASL) [19, 28, 29] and pulse arterial spin labelling (PASL) [30–33]. These approaches are fundamentally different in the extent location and the lasting duration of the labelling plane.

### 4.1. Continuous Arterial Spin Labelling

**Figure 5:**
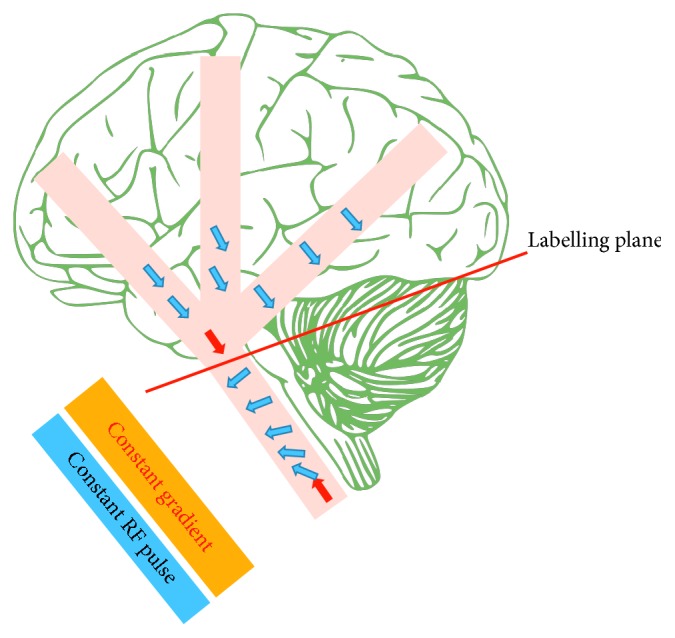
Flow-induced adiabatic inversion. By applying a constant gradient through the flow direction, the flowing blood water gradually changes its local resonance frequency. Simultaneous constant RF pulse is applied over a small spatial location (the labelling plane) where it is on-resonance with the local resonance frequency of the flowing blood water. Subsequently, the flowing blood water inverts as it crosses the labelling plane. Tagged blood water represented by blue arrows and red arrows illustrates the flowing blood water before and after inversion.

In this approach, the flowing blood water is continuously inverted at the location of the feeding artery (neck) among the inversion plane. Here, continuous inversion indicates that the RF pulse lasts for longer than typical RF pulses (around 1 to 3 seconds) [22]. As mentioned previously, the technique of the adiabatic inversion is used in ASL; however, rather than varying RF during the pulse, the motion of the flowing blood water itself under a constant gradient can produce a varying frequency while the RF remains constant, which is referred to as flow-induced adiabatic inversion (Figure 5) [28]. By applying a constant gradient in the flow direction, the local resonance frequency of the flowing blood water is changed. At the same time, a constant RF pulse applied over a small spatial region will invert the flowing blood water when it is on-resonance (its resonant frequency matches that of the RF pulse). This small spatial region is defined as the labelling plane, where the flowing blood water magnetisation inverts as it crosses this plane. This results in a steady stream of tagged blood water while the RF and gradient are switched on.

**Figure 6:**
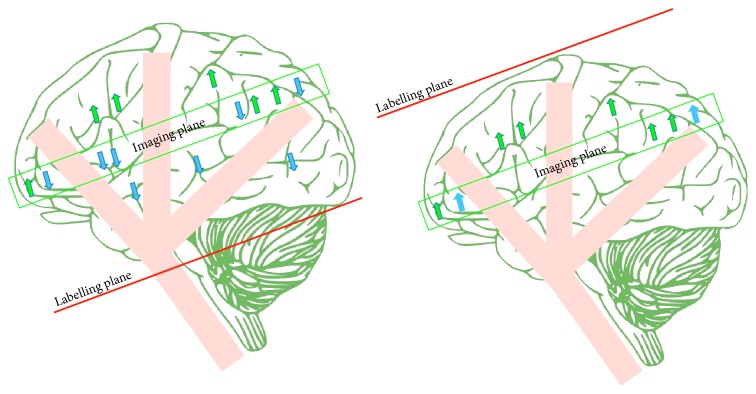
A drawing showing the continuous arterial spin labelling (CASL). Static tissue is shown by green arrows, while tagged blood water is shown by blue arrows.

A typical control image for CASL in early applications was acquired with a labelling plane symmetric to that used in the tagged image but distal to the image plane (Figure 6) in order to compensate for the MT effects [26]. Although this did control MT effects, it effectively controlled only a single image slice. The inversion plane in the control and tagged images requires a symmetrical location around the imaging slice to produce the same amount of MT effects in each, so this solution is not viable for multislice acquisition.

Two techniques have been proposed to control for the problem of MT effects in case of multislice acquisition. The first, proposed by Silva et al., uses two separate RF coils: one to yield a continuous adiabatic inversion (this is a small surface coil for tagging, i.e., placed on the location of the carotid artery) and the other for the image acquisition [34]. This arrangement means that the continuous RF pulse does not affect the image slices by MT effects. It therefore allows the creation of a simple control image without distal inversion from the RF pulse. Moreover, this technique poses another advantage by allowing labelling of one specific artery, which is akin of territory mapping of perfusion [35]. However, this approach produces a high local specific absorption rate (SAR) and requires additional hardware not generally available in MRI scanner setups.

The second technique was therefore proposed by Alsop and Detre to compensate for MT effects in multislice image acquisition. Applying an upstream inversion plane composed of a sine-modulated RF pulse with a gradient identical to the labelling image results in the production of double inversion planes. Subsequently, the flowing spins are inverted through the first plane and un-inverted in the second. Theoretically, this would result in no tagging effect; however, in practice, it produces the same MT effects by using the same overall power as the tagged images. This method suffers from a reduction of tagging efficiency because some of the inverted spins will not encounter the un-inverted zone, and there will therefore be a loss of tagging signal during the subtraction [36].

### 4.2. Pulsed Arterial Spin Labelling

Pulsed arterial spin labelling (PASL) techniques have been developed to deal with the major problems of CASL of high SAR and RF duty-cycle requirement accompanying the use of a continuous RF pulse. Rather than using a continuous RF pulse to invert the flowing spins, PASL applies a slab-selective adiabatic inversion pulse over a thick band that contains the feed arteries and which is proximal to the imaging plane. The image is acquired after a predefined delay, which aims at allowing the tagged water spins to perfuse into the tissue of interest. Collecting high-quality control images is critical in order to provide an accurate ASL difference image. The accurate control images in ASL generally aim to compensate for the MT effects by producing similar static spins to those in the labelling image, with no tagged spins. There are three main PASL sequences, and each applies a different strategy.

**Figure 7:**
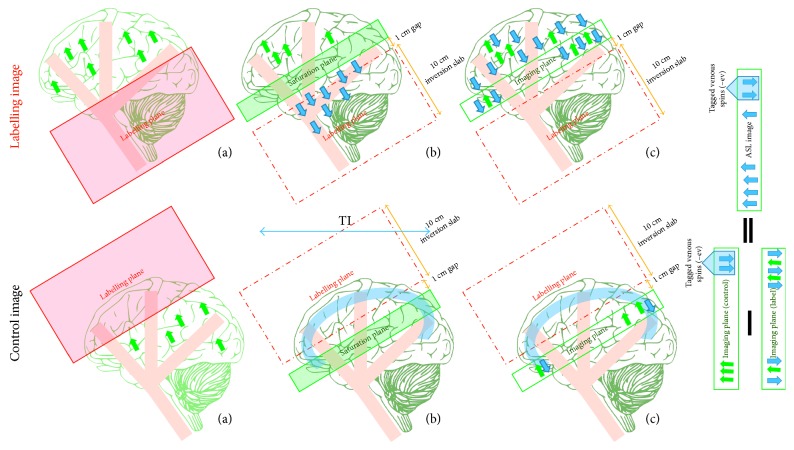
A drawing showing the mechanisms of echo-planar imaging and signal targeting with alternating radiofrequency (EPISTAR). The labelling image is acquired using a large inversion slab (a) during the application of the proximal tagging; (b) a saturation slab is applied to the imaging plane in order to remove tagging contamination; (c) the tagged image is obtained after a delay time (TI) during which the tagged blood water leaves the labelled plane and starts to disperse from the vascular system into tissues at the image plane (arterial arrival time). A control image is acquired using a distal large inversion slab (a) during the application of tagging; (b) then a saturation slab is applied (c) to obtain the control image after the same delay time *TI* where the tagged venous spins enter the control image. The ASL difference image (control−label) includes the tagged venous spins, which are negative and appear as focal dark spots. Static tissue spins are shown as green arrows and tagged blood water is shown as blue arrows.

Echo-planar imaging and signal targeting with alternating radiofrequency (EPISTAR) was the first version of PASL [31]. As shown in Figure 7, the label image is typically acquired using a spatial selective tagging pulse over a 10 cm slab that is 1 cm upstream of the imaging slice. However, due to the small gap used and the fact that the slice profile of the inversion plane is not perfectly rectangular, contamination from the tagging plane may affect the imaging plane. Using a saturation slab on the imaging plane before image acquisition helps us to remove this contamination.

Obtaining a control image presents some difficulties, as in CASL. In order to adjust for the MT effects in the labelled image, an identical inversion slab to that used during labelling can be applied 1 cm distal to the imaging plane. The proximity of this inversion slab to the image edge produces tagged venous blood water that enters the image from above. These labelled venous spins result in focal dark spots in the ASL difference image. Although this method reflects the CBF change, the exact link between the CBF change and the alteration in the signal intensity is unknown. In addition to these drawback of EPISTAR, it is more sensitive to the *T*1 relaxation time of the blood (*T*1*a*) because the tagging is lost during *T*1*a* while the flowing spins travel to the imaging slice [31].

**Figure 8:**
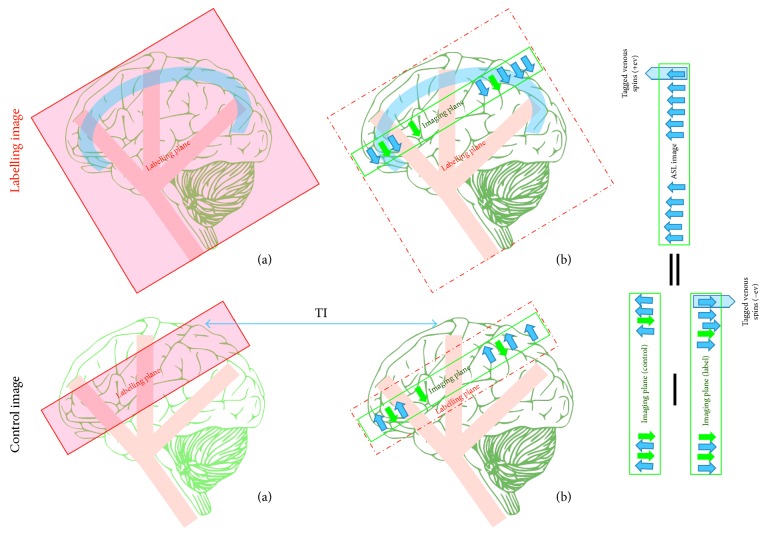
A drawing showing the flow-sensitive inversion pulse (FAIR) mechanism. A labelling image is acquired using a large inversion slab, (a) during application of the nonselective slice pulse; (b) the tagged image is obtained after a delay time (*TI*) and contains tagged flowing spins from arteries and veins. A control image is acquired using the same inversion pulse in a slice-selective (a). The slice selected is larger than the image slice (b). The control image is obtained after *TI*. The ASL difference image (control−label) shows the tagged venous spins as bright signals. Static tissue spins are shown as green arrows and tagged blood water as blue arrows.

To overcome the aforementioned problems of EPISTAR, Kim introduced another PASL strategy in 1995 [32]. The technique is called flow-sensitive inversion pulse (FAIR), and it works by subtracting two inversion images, one with and one without a slice-selective inversion pulse (Figure 8). The tag image is obtained after a delay time with a nonslice-selective inversion pulse. As a result, all the spins inside and outside of the image plane will be inverted, and the spatial extent of the RF pulse will be as large as the physical RF coil. This has the advantage of tagging the blood flow entering the imaging slice from multiple directions; however, it also tags venous blood flow. The inverted venous spins enter the tagged image and produces artefacts that appear as bright signals in the difference image, rather than dark spots as in the EPISTAR technique. Moreover, there is no need for a saturation pulse before image acquisition, as there is no gap between the label plane and the image slice.

The control image is acquired using the slice-selective inversion pulse, which is the same as that in the label image. Thus, as in the entry slice phenomena, the inverted blood water is replaced by totally relaxed spins due to blood flow, while the static spins will remain inverted. As the same inversion pulse is used in both control and tag images, the MT effects tend to cancel out. The slice-selective profile is not perfectly rectangular, so it must be larger than the image slice. The image is thereby obtained at the centre of the slice-selective inversion where clean uniform inversions cover the entire image slice [32, 37].

**Figure 9:**
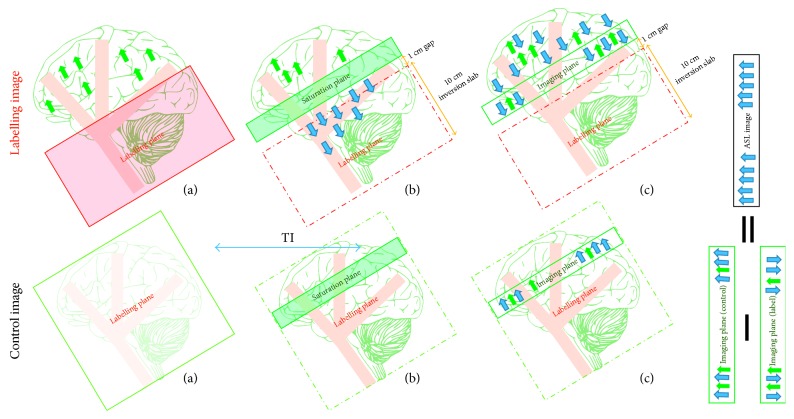
A drawing showing the mechanism for proximal inversion with a control for off-resonance effect (PICORE) mechanism. The labelling image is acquired using a large inversion slab (a) during application of proximal tagging; (b) a saturation slab is placed on the imaging plane in order to remove tagging contamination; (c) the tagged image is obtained after a delay time (*TI*) where the tagged blood water leaves the labelled plane and starts diffusing from intravascular vessels into tissue at the image plane during arterial arrival time. The control image is acquired using (a) a shifted RF pulse with no gradient; (b) a saturation slab followed by (c) obtains the control image after the same delay time *TI*. The ASL difference image (control−label), unlike those in EPISTAR and FAIR, does not involve any tagged venous spins. Static tissue is shown using green arrows and tagged blood water with blue arrows.

A third PASL technique that derives from the EPISTAR method is proximal inversion with a control for off-resonance effect (PICORE). This method uses an identical tag image to that used in EPISTAR [33] (Figure 9). However, the control image is acquired using a shifted RF pulse (off-resonance) with no gradient. Thus, they have the same offset that the tagged image experiences, and hence, the control image will produce the same MT effects without any inversion. Since there is no tagging in the control image, it takes advantages of avoiding tagging the venous blood flow that enters the image slice from the distal end in contrast to EPISTAR and FAIR.

As seen previously, these techniques differ in terms of sensitivity to the inflow from the distal end of the image slice, tag profile, and control acquisition, where choice of one approach over the other depends on the blood supply geometry. If the blood supply is entering the slice of interest form known direction, then PICORE approach will be in favour because it does no produce any venous tagging. However, in case of an unknown direction of blood supply, such as in a watershed area (the region that receives dual blood supply from furthest distal arterioles of two large arteries), FAIR would be the more conservative approach because it ensures that all the arterial blood entering the slice of interest will be tagged. From a labelling viewpoint, PICORE and FAIR are superior to EPISTAR, but EPISTAR has the advantage of lower level of eddy current artefact because it uses identical gradient selection for the labelling plane in both the control and tagged images and hence balance gradient.

A further consideration that affects the selection of one tagging approach over another is the inversion slice profile. To clarify the effect of the inversion slice profile, slice thickness must be explained first. Back to the basics of the slice selection where the thin slice is achieved using a steep gradient that results in a large frequency difference between two points, the range of the frequencies between these points is called the bandwidth. Beside the steep gradient, a transmitted RF pulse that matches the point of difference is used, and this is known as the transmit bandwidth [38]. This RF pulse will impact the narrow band of frequencies and hence sharp slice profile fulfilled, and vice versa for thick slice. In terms of the effects of the inversion slice profile on the preference of the tagging method, FAIR, unlike EPISTAR or PICORE, in the case of single slice imaging, uses thin inversion slice and hence sharp cutoff that is beneficial in CBF quantification [33]. However, this is not true in case of multislice or large imaging of 3D volume.

### 4.3. Pseudocontinuous Arterial Spin Labelling

Recently, a modified version of CASL which overcomes most of the original technique's weaknesses has emerged; this is known as pseudocontinuous arterial spin labelling (PCASL) [29]. This method mimics the effect of CASL by using a long series of RF pulses rather than a continuous RF pulse. However, this results in an aliased tag plane that can be removed by making the RF pulse more selective. This is achieved by using a strong gradient and short-spaced RF pulses. This reduces the error of phase tracking between the RF pulses and the flowing spins and hence increases labelling efficiency.

**Figure 10:**
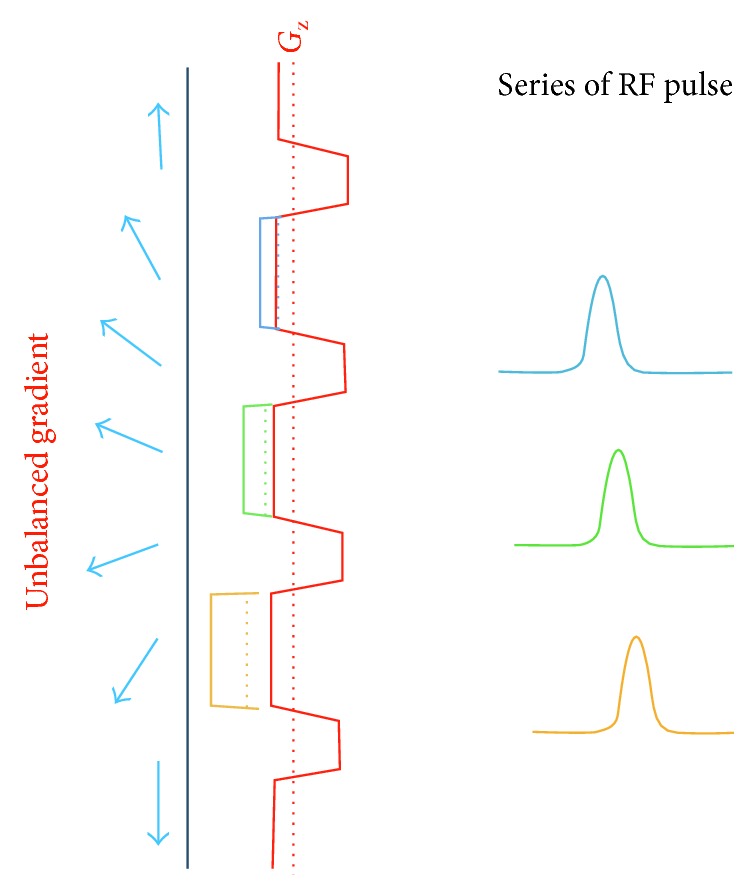
A drawing showing the composition of the labelling plane of pseudocontinuous arterial spin labelling (PCASL). The unbalanced gradient produce ≈1 mT/m on average and the RF pulses also produce a B1 ≈ 1 mT/m on average. The blue arrows represent the flowing blood water before and after inversion. Different colour gradients and RF pulses represent the amplitude increases and the adjusted RF pulse phases, respectively.

**Figure 11:**
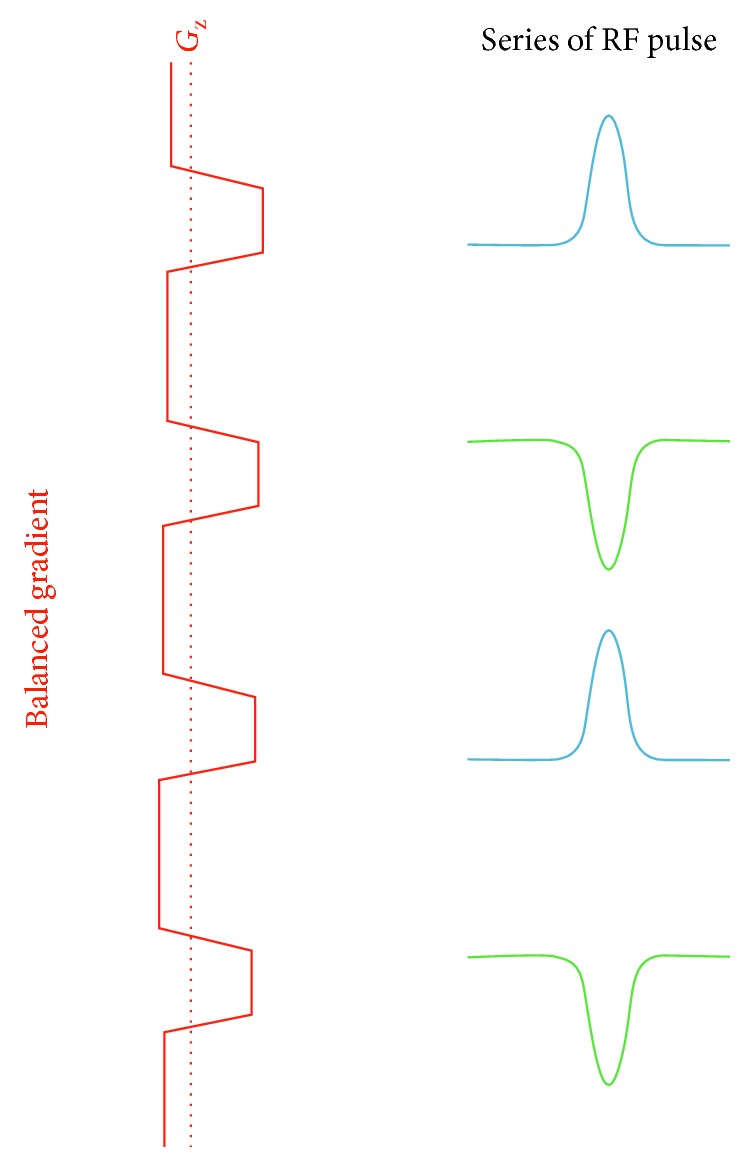
A drawing showing the composition of the control pulse sequence of pseudocontinuous arterial spin labelling (PCASL). The balanced gradient produces zero on average, and the alternating RF pulses produce a B1 that is also zero on average. The blue and green RF pulses represent the alternative behaviours that result in a zero-average B1.

On the tag plane, a pulsed RF and a strong slice-selective gradient are used with an amplitude of approximately 10 times than that used in CASL. As shows in Figure 10, the refocusing portion of the slice selection gradient is increased in amplitude (unbalanced) so that the flowing spins accumulate the additional phase. In order to adjust the RF pulse's phase to become in-resonance with the flowing spin's accumulated phase, the *n* RF pulse phase (Φ*n*) should be Φ*n* = *γ* · *n* · *G* · *T* · *Z*, where *γ* is the gyromagnetic ratio, *G* is the average gradient (≈1 mT/m in the labelling plane), *T* is the spacing of the RF pulses, and *Z* is the distance from the gradient isocentre to the particular point at which the gradient pulse of the labelling plane is applied. On the other hand, the slice selection gradient is balanced among the control image with a series of alternating RF pulses (Figure 11). These produce a zero-average gradient between each pair of RF pulses as well as a zero-average B1. Although this provides no tagging, it creates the same RF power as that used in the labelling image and hence creates the same MT effects [29].

**Figure 12:**
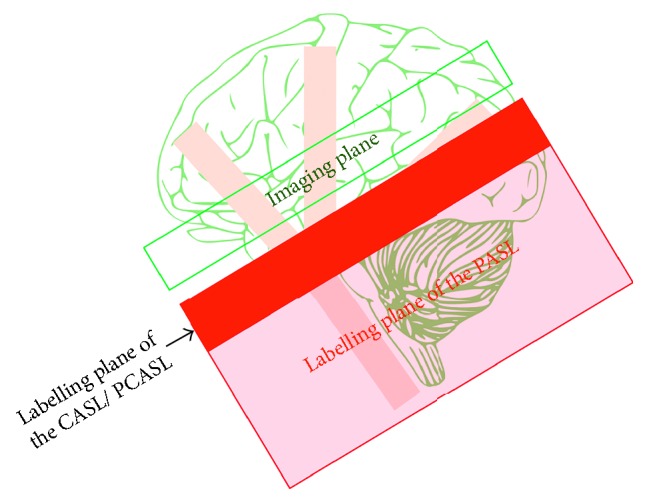
A drawing showing the difference between the tag plane of the CASL/PCASL and that of PASL.

PCASL offers advantages over PASL because it produces a higher SNR than that yielded by PASL. This occurs for two reasons: first, PCASL and CASL have longer labelling durations than PASL, so they each provide a larger tagged blood volume with a higher SNR in consequence; second, both CASL/PCASL and PASL have spatial gaps between the label and image planes; this gap (the transition zone width) is larger for PASL because the label plane of the CASL/PCASL is located at the distal end unlike that in PASL (Figure 12). PASL also simultaneously inverts the whole slab unlike the CASL/PCASL, which tags the flowing blood as it crosses the tag plane. In other words, tagged blood from the CASL/PCASL takes less time to travel from the tagging slab to the image of interest (the transition zone width). As a result, CASL/PCASL will suffer less *T*1 decay and thus offer higher SNR than PASL [22].

PCASL also surpasses CASL due to two additional advantages. MT effects are almost entirely eliminated through PCASL, while the long RF pulse used in CASL results in additional MT effects and can promote subtraction errors. The solutions that are offered in response to this issue can also lead to a reduction in labelling efficiency. In contrast, PCASL utilises a strong gradient during the application of the RF pulses that results in the RF pulses becoming further off-resonance relative to the imaging region. This effectively reduces the MT effects and enhances labelling efficiency. CASL also requires application of a continuous RF pulse that is particularly short and high powered. This pulse type requires a modified RF amplifier that is not available in most current clinical imaging systems. In comparison, PCASL RF pulses can be implemented on present RF amplifier systems [22, 39]. Thus, PCASL won the preference and is recommended by the white paper [22] (it is a summary for the recommended ASL implementation with the consensus of the International Society for Magnetic Resonance in Medicine (ISMRM) and the European ASL in dementia consortium).

## 5. Technical Considerations for CBF Quantification

ASL image quality is affected by several intrinsic parameters (physical and physiological) that can confuse the relationship between the obtained signal and the perfusion value. These intrinsic parameters should be considered and understood in order to select the most appropriate extrinsic parameters and labelling approach. These intrinsic parameters include MT effects, transit delay (flow velocity dependent), local *T*1 relaxation time, arterial bolus width, and labelling efficiency. MT effects have been previously mentioned, and ways to adjust the procedures to minimise these effects have been discussed for each labelling approach.

**Figure 13:**
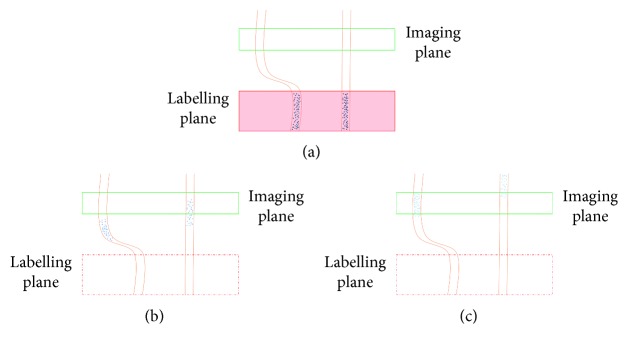
A drawing showing various arterial arrival times depending on the vessel structure (a) during labelling, (b) during the first transit time after labelling, and (c) during the second transit time following labelling. Notice how the tagged bolus loses tagging via *T*1 decay, which is displayed as a lightening of shade.

Arterial transit time or arrival time refers to the time taken for the labelled blood water to reach the imaging plane. Transition of the tagged blood water through the vessels varies depending on the vessels' size, length, and tortuosity (Figure 13). ASL signals may vary in intensity due to this variety of transit times even when they provide the same blood perfusion. In order to reduce the sensitivity of CBF quantification to transit time, Alsop and Detre introduced the idea of a postlabelling delay (PLD) [40] to allow the tagged bolus to reach the imaging plane. As the tagged bolus travels to the tissue of interest, it loses some tagging via *T*1 decay (Figure 13). Moreover, both transit time and *T*1 decay are on the order of seconds, so *T*1 and transit time are competing constant times that should be considered when determining the PLD.

To add a delay to the pulse sequence and allow all the tagged spins to arrive, the duration of the arterial bolus must be controlled. This duration (bolus width) is defined as the total time required for all the tagged blood water to leave the tagging plane. In CASL, the duration of the arterial bolus is well defined, being equal to the RF pulse duration. In this case, the tagging band consists of a thin line with continuous labelling (long RF pulse) where the flowing spins are inverted as they cross this line. Thus, once the RF pulse stops, all the tagged blood leaves this labelling line. Consequently, if the CBF increases, there is also an increase in the amount of the tagged blood water crossing the labelling plane, which is reflected on the ASL image as an increase of the signal intensity; the opposite reaction is seen with a decrease in the CBF.

**Figure 14:**
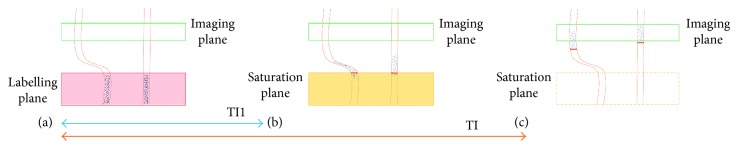
This drawing shows the QUIPSS II technique and its particular conditions. The first condition is satisfied (a-b) *TI*1 < tagged bolus, and the second condition is that *TI* − *TI*1 > the transit delay.

On the other hand, in PASL, the duration of the arterial bolus is poorly defined (ill-defined). The tagging band is over a specific volume with short duration. It produces a fix volume of the tagging blood water regardless of any increase or decrease in the CBF. In order to distinguish between high and low CBF, a saturation pulse can be applied over the labelling plan after blood water labelling (*TI*1) to cutoff the tagging tail (Figure 14). This produces a defined tagged bolus in time rather space. Thus, if the CBF increases, the volume of labelled blood also increases, leaving the tagging volume as that produced before the cutoff. This technique is known as quantitative image of perfusion using a single subtraction (QUIPSS II) [41]. For QUIPSS II to yield a quantitative flow image, two conditions must be satisfied: (1) the saturation pulse must be applied before all of the tagged spins have left the tagging band so that *TI*1<tagged bolus; (2) the delay after the saturation pulse must be long enough for all the arterial tagged spins to reach the tissue voxel so that *TI* − *TI*1 > the transit delay. A similar technique used to improve the accuracy of the perfusion measurement is known as QUIPSS II with thin slice *TI*1 periodic saturation (Q2TIPS) [42]. It replaces the single saturation pulse with a thin slice periodic train of saturation pulses at the distal end of the tagging slab.

The difference in arterial blood magnetisation immediately after a perfect inversion pulse is 2*M*_0_*a*. However, this is not the case as the ASL tagging approaches have some inversion deficiency. The ratio between the actual ASL difference image with imperfect inversion to the perfect 2*M*_0_*a* is referred to as the labelling efficiency, *α* [43, 44]. Each labelling approach offers a different ratio of labelling efficiency, which can be taken into account during the calculation of the CBF.

## 6. Improving the SNR

Image quality increases as the SNR increases, yet ASL images suffer from poor SNR as an inherent problem. Usually in MRI, the SNR is increased by strengthening the signals received and/or reducing the noise. This can be taken into account in ASL by considering the SNR impact factors including the hardware used, acquisition parameters, and the application of special techniques.

### 6.1. Hardware Considerations

In MRI, a high magnetic field results in signals quadratically increases while the noise increases linearly [45]. Acquiring ASL images under a high magnetic field will therefore elevate the SNR because it produces high magnetisation as well as lengthening the *T*1 relaxation time of blood [46]. However, this benefit comes with some disadvantages. The magnetic field's homogeneity decreases as its strength increases; additionally, elevated field strength coexists with B1 increase and the subsequent increase of RF power deposition. Despite these disadvantages, a high magnetic field can still be useful to overcome low field strength limitations [47].

Coil type also affects the received signals' quality and strength. Multichannel receiver coils are recommended [22] because these allow data to be received from several channels and hence boost ASL SNR. In addition, they enable acceleration of the imaging using parallel imaging.

### 6.2. Acquisition Parameters and Special Techniques

Generally, a large voxel size results in a high SNR because the large voxel contains more protons and provides stronger signals. However, it reduces the spatial resolution, can cause blurring of the image details, and introduces a partial volume effect. This effect among the ASL images causes perfusion cross-contamination, which can result in underestimation of the grey matter (GM) and overestimation of the white matter (WM) perfusion [48]. The reason for this shortcoming is the lower blood flow and longer transit time of the blood in the WM compared to the GM; notably, the perfusion in WM ranges from one-tenth to one-third of the GM values. As a result, WM produces lower perfusion signals than GM. The recommended voxel size is therefore 3 to 4 mm inplane and 4 to 8 mm through plane [22] as a compromise between improved SNR and the required image clarity.

Increasing the averaging number by acquiring repeated control and label images can also strengthen ASL signals. However, this lengthens the scan time and introduces motion artefacts. The lengthened scan time can be reduced and hence the motion artefact by using parallel acceleration via undersampling the K space, but as this acceleration can limit the SNR, a moderate acceleration is recommended, with a factor of 2 to 3 [22]. The introduced artefacts can be overcome during acquisition via background suppression and by acquiring the repeated images in an interleaved pattern. This pattern helps us to reduce the misregistration artefact that may be introduced during image subtraction, and the remaining motion artefact can be further reduced during preprocessing via coregistration.

As previously mentioned, ASL signals improve through the use of low spatial resolution (large voxel) and increase of the averaging. However, these processes result in signal fluctuation due to physiological noise and motion artefact. Static tissues are dominating the source of the noise and artefact. Thus, the SNR can be elevated through reduction of the static tissue using inversion pulses after the labelling pulse which is referred to background suppression (BS) [50, 51]. Here, it is important to notice that after the labelling pulse, the inverted spins go back to equilibrium with *T*1 time constant, as well as the difference value (control−label) decays toward zero in the same time constant. Thus, the BS is applied globally and hence does not affect the difference value. Nevertheless, typical BS efficiency is 95%, which means that every inversion pulse used will distort the ASL signals by approximately 5% [51]. Moreover, using BS in the multislice imaging method or the methods that use multiexcitation pulses per repetition time TR will have different effectiveness levels across the slices. In contrast, this technique is more efficient among the imaging methods that are using one excitation per TR such as single slice 2D, single-shot 3D, or segmented 3D. In terms of segmented 3D imaging methods, the technique remains important; however, not only to enhance the SNR but to remove the ghosting artefact (motion artefact resulting from phase inconsistencies between the segments).

ASL image quality is most significantly affected by the volume of labelled blood water reaching the extravascular space. As this volume increases, ASL SNR increases. In CASL/PCASL methods, this volume is determined by the labelling duration, which in turn is limited by the *T*1 relaxation of brain tissue, especially *T*1*a*-relaxation. Labelling duration much longer than *T*1*a* relaxation will be with diminishing returns since the advantage of adding newly labelled blood will be offset by the loss of signal due to *T*1*a*-relaxation from earlier spin labelling. The corresponding loss of SNR and long TR would require more image averages to obtain the same SNR, making the experiment impractical. A labelling duration of 1800 ms has been recommended as a compromise between increasing the SNR, power deposition, and to preserve the SNR in case of long ATT, whilst balancing loss of signal due to *T*1*a* decays at 3T [22].

On the other hand, the labelled bolus volume of the PASL is determined by the labelling slab size, and hence this should be kept as large as possible. However, the labelling slab thickness is limited by three factors. Increasing the slab thickness means increasing the transition zone width, and during this long transition, the tagged spins will lose signal. The size of the RF transmit coil also limits the possible slab thickness. Finally, B1 from the RF transmit coil falls of far from the isocentre, so the blood water at the coil edges will only be partially inverted, and this will affect labelling efficiency. Additionally, these partially inverted spins will need a longer time to washout from the labelling slab. This washout necessitates a long TR before the next tagging pulse, which reduces the time efficiency. A slab thickness of 15–20 cm has been recommended to balance these factors.

## 7. Further Parameters beyond the CBF

Acquiring the ASL images at single time point provides absolute quantification, but this method is sensitive to transit time. As aforementioned, this sensitivity can be reduced by using a postlabelling delay in CASL/PCASL or by using QUIPSS II/Q2TIPS in PASL. Additional perfusion parameters can also be extracted from the ASL images. By collecting the ASL images at multiple delay time points, information about the flow and the labelling delay times can be gathered. In this way, it becomes possible to measure the transit time and the bolus duration beside the CBF [52–56] by fitting the acquired images at multiple time points to the general kinetic model.

In addition, using ASL model-free allows measurement of arterial blood volume (aBV) [57] by acquiring the ASL image at multiple time points with and without a crusher gradient. This enables estimation of the local arterial input function (AIF) by subtraction of the acquired perfusion images with and without the crusher. Consequently, deconvolution of the tissue perfusion signals curve with the AIF curve can provide the residue function (the amplitude of which simply reflects the CBF), while the aBV can be estimated by integration of the AIFs.

## 8. Conclusion

ASL advantages compared to other MRI perfusion methods, include its noninvasive character, no need for exogenous contrast agent, and absolute CBF quantification. Despite these remarkable advantages, ASL has not yet replaced the other invasive MR perfusion modalities on a widescale. This is mostly due to the low SNR. This makes the method ideal for vulnerable patient populations including children, posttreatment oncological patients who have no tolerance for high-rate contrast injections and difficult venous access, and patients suffering from renal insufficiency.
